# Human Exposure Pathways to Per- and Polyfluoroalkyl Substances (PFASs)—A Comprehensive Review of Sources, Physicochemical Properties, and Human Health Risk Assessment

**DOI:** 10.3390/toxics14060528

**Published:** 2026-06-18

**Authors:** Andrzej R. Reindl, Jakub A. Zduńczuk

**Affiliations:** Department of Environmental Toxicology, Faculty of Health Sciences, Medical University of Gdansk, 80-210 Gdansk, Poland; jakubzdunczuk@gumed.edu.pl

**Keywords:** PFAS, human exposure, forever chemicals, environmental persistence, bioaccumulation, regulatory framework, occupational health

## Abstract

Per- and polyfluoroalkyl substances (PFASs) present a critical challenge to global public health and environmental integrity due to the exceptional stability of the carbon–fluorine (C–F) bond. This review synthesizes current knowledge on PFAS physicochemical properties, exposure pathways, and toxicological outcomes, while evaluating global regulatory efficacy. A central problem addressed in this review is the widening discrepancy between rigid, yet deeply fragmented, international regulatory frameworks and the increasingly complex, non-linear epidemiological data regarding PFAS health risks. While historical paradigms focused heavily on direct carcinogenicity, recent high-resolution data reveal significant heterogeneity and methodological inconsistencies in cancer links. Instead, robust evidence points to severe systemic toxicities—including hepatotoxicity, immunotoxicity, and maternal–fetal disruptions—frequently driven by mixture co-exposures and sex-specific metabolic dimorphisms. Furthermore, the industrial transition to short-chain substitutes has inadvertently compounded the crisis due to their high environmental mobility and resistance to conventional water treatment. By critically evaluating these toxicological and regulatory contradictions, this review demonstrates that current substance-by-substance legislative models fail to mitigate real-world pollution trends. Ultimately, we emphasize the urgent need to transition to holistic mixture modeling, implement unified class-based global regulations, and accelerate advanced destructive remediation technologies to mineralize the resilient C–F bond.

## 1. Introduction

Per- and polyfluoroalkyl substances (PFASs) constitute a vast group of thousands of synthetic organic chemicals characterized by the presence of at least one fully fluorinated carbon atom. This structural feature, where hydrogen atoms bonded to carbon are replaced by fluorine, defines the unique chemical identity of this group [1].

PFASs have been integrated into various industrial sectors for decades, valued for their exceptional thermal stability and surfactant properties. Since the 1940s, they have been utilized in the production of non-stick coatings for packaging and cookware, protective clothing, aqueous film-forming foams (AFFFs), electronics, semiconductors, and numerous other consumer products [1,2].

The most distinctive feature of PFASs is their extraordinary stability, stemming from the high-energy C–F bonds. These bonds provide chemical and thermal resistance, rendering the molecules virtually immune to both biotic and abiotic degradation in the environment. Consequently, mitigating PFAS pollution is a complex challenge exacerbated by a significant lack of effective large-scale neutralization methods [1].

Growing environmental evidence confirms the ubiquitous presence of PFASs in natural ecosystems and their ability to migrate across environmental media, persisting for decades. Studies have documented PFASs in wildlife, surface waters, sediments, and the atmosphere. Notably, contamination is found even in remote regions lacking local emission sources, proving the capacity of these compounds for long-range transport [1,3].

Current toxicological research correlates PFAS exposure with significant health risks, including increased incidences of cancer, infertility, liver and kidney diseases, and disruptions of the cardiovascular and immune systems [4]. Humans are exposed to PFASs through occupational and lifestyle pathways, including inhalation, ingestion (contaminated water and food), and dermal contact with consumer products [2]. Due to their persistence and bioaccumulative nature, PFASs represent a critical public health challenge, prompting a surge in scientific interest and an increasingly stringent global regulatory landscape [1,5]. This review analyzes current knowledge regarding exposure sources and evaluates regulatory efficacy based on global pollution trends.

Given their persistence, bioaccumulative potential, and widespread emissions, PFASs represent a major public health challenge. The increasing volume of research documenting their toxicity has prompted regulatory bodies worldwide to implement stricter legal frameworks [1,5]. Understanding the physicochemical properties and primary exposure routes is essential for preventing further contamination and developing effective remediation strategies. This review aims to synthesize current knowledge on PFAS exposure sources and evaluate the efficacy of existing regulations based on observed pollution trends.

## 2. Characteristics of Per- and Polyfluoroalkyl Substances

### 2.1. Historical Context and Origin

The history of PFASs is rooted in the rapid advancement of organofluorine chemistry during the first half of the 20th century. A pivotal moment occurred in 1938 when Dr. Roy Plunkett, working for DuPont, discovered polytetrafluoroethylene (PTFE) while researching refrigerant gases. This material, branded as Teflon, exhibited unprecedented thermal resistance, low friction, and chemical inertness. Its utility was proven during World War II within the Manhattan Project, which catalyzed the industrial expansion of fluoropolymer technologies [6].

Commercialization intensified in the 1950s, leading to the synthesis of other PFASs such as perfluorooctane sulfonic acid (PFOS) and perfluorooctanoic acid (PFOA). Their unique ability to repel both water and oil made them indispensable for surface treatments and fire suppression [6,7]. However, the same properties that ensured industrial success led to global environmental persistence. After 2000, the recognition of their bioaccumulative nature and health risks led to the tightening of regulations, particularly for long-chain PFASs, and the formalization of definitions by organizations like the OECD [6,7].

### 2.2. Definitions and Nomenclature

The 2021 OECD definition classifies PFASs as organic compounds containing at least one fully fluorinated methyl (–CF3) or methylene (–CF2–) group. This updated definition is more inclusive than the 2011 Buck et al. criteria, which focused primarily on perfluoroalkyl moieties (C_n_F2_n+1_–) [1,8]. This shift reflects the complexity of the PFAS landscape; while a 2018 report cataloged 4730 PFASs, the 2021 criteria expanded this number to over 7 million potential substances in the PubChem database. Such diversity underscores the need for standardized nomenclature to facilitate effective monitoring and regulation [1,8,9].

### 2.3. Classification

The primary distinction lies between perfluoroalkyl substances (where all C–H bonds in the alkyl chain are replaced by C–F bonds) and polifluoroalkyl substances (where at least one C–H bond remains). Polyfluoroalkyl compounds often serve as precursors, degrading in the environment into highly stable perfluoroalkyl “terminal” products [1]. PFASs are further categorized by functional groups, such as perfluoroalkyl carboxylic acids (PFCAs, e.g., PFOA) and perfluoroalkyl sulfonic acids (PFSAs, e.g., PFOS). Furthermore, chain length is a critical factor: long-chain PFASs (PFCAs ≥ C7, PFSAs ≥ C6) are more bioaccumulative, while short-chain alternatives, introduced as safer substitutes, exhibit higher environmental mobility and different toxicological profiles [1,4,10].

### 2.4. Physicochemical Properties

The unique properties of PFASs determine both their industrial utility and the difficulty of environmental remediation. The C–F bond, one of the strongest in organic chemistry (~485 kJ/mol), provides resistance to hydrolysis, photolysis, and biodegradation, earning them the name “Forever Chemicals” [1,11,12]. Their amphiphilic nature allows them to repel both water and oil, making them superior surfactants compared to hydrocarbon-based alternatives [2,4,13]. Solubility is inversely proportional to chain length, dictating their distribution between aqueous phases and solid matrices like soil or sediment [14]. The specific physicochemical parameters of the most widely studied PFASs, namely PFOA and PFOS, are summarized in Table 1.

### 2.5. Environmental Persistence and Toxicokinetics

PFASs resist natural degradation, leading to continuous accumulation since the 1950s [10,14]. Short-chain compounds are highly mobile in aqueous systems, while long-chain compounds adsorb strongly to soil particles and bottom sediments [10,14]. Both undergo long-range atmospheric and oceanic transport, reaching remote indicator areas like Antarctica, where they are detected even in penguins [3,10]. Unlike classic POPs that accumulate in lipids, PFASs bind to transport proteins such as serum albumin and fatty acid-binding proteins (FABPs), concentrating in the liver, kidneys, and blood [15,16]. They exhibit trophic magnification, with levels increasing toward apex predators [17]. Toxicological effects include PPAR activation, oxidative stress, and endocrine disruption, with long-chain PFASs exhibiting potential carcinogenic effects, as well as reproductive and developmental toxicity [4,10,18,19,20]. Human elimination half-lives are exceptionally long: ~3.8 years for PFOA, 5.4 years for PFOS, and up to 8.5 years for PFHxS [4,10].

## 3. Human Exposure Pathways

### 3.1. Industrial and Consumer Sources

Widespread global PFAS production and use in over 200 OECD categories explain their ubiquity, with historical emissions of long-chain PFCAs estimated at 2610–21,400 tons [13,21,22]. Primary emissions stem from AFFFs at military and civilian airports, firefighting training centers, and petrochemical facilities (such as oil refineries) as well as industrial discharges from fluorochemical, textile, and paper plants [4,13,23,24,25]. Consumer exposure occurs via migration from food contact materials (FCMs), non-stick cookware (particularly when overheated >260 °C) [12], and indoor dust [26,27]. Protective masks also constitute a relevant consumer product source of exposure [28]. The primary sectors of PFAS application and their corresponding human exposure routes are summarized in Table 2.

### 3.2. Drinking Water Exposure

PFASs contaminate groundwater via point sources, such as AFFF training sites, and diffuse sources, including the application of biosolids on agricultural land [25]. In the US, PFASs are detected in ~45% of tap water samples [33]. In Europe, approximately 23,000 contaminated sites have been identified, including 2300 hotspots [34,35]. Conventional water treatment is ineffective; advanced remediation (GAC, reverse osmosis) is required but shows diminishing efficacy for short-chain compounds [14,36]. Bottled water does not automatically represent a safe alternative, as PFASs can also be present in these products [36,37]. Global regulatory bodies have established varying thresholds for PFASs in drinking water to mitigate these risks [35,37,38,39,40], as compared in Table 3.

### 3.3. Dietary Exposure

Dietary intake via aquatic, plant, and animal-derived products constitutes a major human exposure pathway [44,45]. Ingestion of contaminated seafood is highly significant due to protein binding in fish liver and serum [15,16], with PFOS exhibiting a higher trophic magnification factor (TMF) than PFOA [17]. Migration of up to 68 different PFASs from paper and cardboard packaging also contributes significantly, influenced by contact time and fat content [29,30]. Furthermore, PFAS accumulation in crops (e.g., leafy vegetables, strawberries) grown in biosolid-treated soil [5,25,44], and their subsequent transfer to meat, milk, and eggs, integrates these chemicals deeply into the human food chain [46].

### 3.4. Air and Dust Inhalation

Indoor dust serves as a primary reservoir for PFASs migrating from treated furniture, building materials, and carpets [26]. Profiles vary by building age; older buildings are dominated by PFOS, while newer ones show short-chain PFASs like PFBS [26,27]. Children are at higher risk due to hand-to-mouth behavior and proximity to floors, often exhibiting higher serum levels than their mothers [47]. Industrial emissions, such as the estimated 0.4–1.3 tons of PFOA emitted annually from a Chinese industrial park, facilitate regional and global transport at altitudes above 1500 m [48,49]. This atmospheric transport drives contamination in remote regions like the Arctic (alert station, 82° N), where monitoring (2014–2023) shows resurgent levels of short-chain PFASs like TFA and PFBA [25,48,49,50].

### 3.5. Dermal Exposure and Absorption

Dermal exposure occurs primarily through cosmetics and DWR-treated clothing [31,32]. In a study of 231 cosmetics from the US and Canada, over half contained high fluorine levels, yet under 8% listed PFASs on labels; concentrations in highly fluorinated products ranged from 22 to 10,500 ng/g [31]. Estimated dermal exposure from cosmetics can reach a median of 1.28 ng/kg bw/day, which may independently exceed EFSA’s recommended limits [51]. Direct, prolonged contact with DWR-treated outdoor textiles provides another consistent exposure route [32].

While initial assessments frequently focused on the deposition of PFASs on the skin surface, recent evidence confirms that these compounds actively penetrate through the dermal barrier to enter the systemic circulation. The transdermal permeation efficiency of PFASs is heavily dictated by their physicochemical properties, particularly the carbon chain length and lipophilicity. Short-chain PFASs demonstrate substantial dermal bioavailability. In in vitro 3D human skin equivalent (3D-HSE) models exposed for 36 h, the absorbed fractions of perfluoropentanoic acid (PFPeA) and perfluorobutane sulfonate (PFBS) reached 58.9% and 48.7%, respectively [52]. Similarly, when human skin models were exposed to PFAS mixtures for 24 h, only short-chain compounds (such as FBSA, PFBA, PFPrS, and PFPeA) successfully penetrated into the receptor fluid [53]. Conversely, dermal permeation decreases significantly as the carbon chain length increases. For the legacy long-chain compound perfluorooctanoic acid (PFOA), the absorbed fraction in 3D-HSE models was 13.5% after 36 h [52], whereas other rigorous evaluations recommend a more conservative fractional absorption value of 1.6% for finite dose human exposure assessments [54].

Crucially, the absence of immediate systemic permeation for long-chain PFASs does not eliminate their toxicological risk, primarily due to the skin reservoir effect. Highly lipophilic, long-chain PFASs (Cn ≥ 9) exhibit a strong affinity for skin tissue and accumulate within the epidermal layers. Studies have shown that up to 66.5% of applied perfluoroundecanoic acid (PFUnDA) and 68.3% of perfluorononane sulfonate (PFNS) are retained directly within the skin tissue, with no immediate detection in the absorbed fluid fraction [52]. Ex vivo diffusion studies on porcine skin also indicate that a majority of applied PFOA and PFBA is retained in the upper skin layers rather than permeating entirely [55]. This tissue retention creates a localized reservoir from which PFASs are continuously and slowly released into the systemic circulation. In in vivo rat models, the peak times of PFASs in systemic circulation following dermal exposure were found to be between 8 and 72 h—markedly longer than the 1 to 24 h observed following oral administration—which highlights the prolonged nature of dermal penetration [56].

Once absorbed systemically, dermally applied PFASs induce significant toxicological outcomes. Subchronic topical exposure to PFHpS and PFOS in murine models successfully elevated serum and urine concentrations, triggering hepatotoxicity (increased relative liver weight) and marked immunosuppression, including reduced B-cells and macrophages [57]. Sublethal developmental effects from dermal contact have also been documented in postmetamorphic amphibians at lowest-observable-effect concentrations (LOEC) of 50 to 120 ppb [58]. Additionally, emerging predictive models suggest that neutral PFASs can undergo direct transdermal absorption from the gas phase, potentially making indoor air a more significant dermal exposure pathway than inhalation for certain compounds [59].

To systematize the kinetic variables driving transdermal penetration and tissue retention, key absorption parameters for selected PFASs are summarized in Table 4.

### 3.6. Occupational Hazards

Human exposure to PFASs exhibits significant regional and demographic variability, revealing profound disparities between the general public and highly exposed cohorts. Large-scale biomonitoring in the United States (NHANES 1999–2018) demonstrates that factors such as age, sex, and race or ethnicity strongly influence serum PFAS concentrations, with adult males and specific racial groups exhibiting higher exposure risks [60]. Furthermore, spatial mapping of pregnant women in the US indicates that geographic location and proximity to local point sources can often overshadow expected demographic trends, highlighting critical environmental justice considerations regarding vulnerable populations [61]. In Europe, the HBM4EU biomonitoring initiative revealed that 14.3% of teenagers overall, and up to 24% in Northern and Western Europe, possessed serum PFAS levels exceeding the health-based guidance equivalent of 6.9 μg/L established by EFSA [62].

These background population burdens contrast sharply with the extreme concentrations identified in environmental hotspots and occupational environments. For instance, the HBM4EU study recorded 95th percentile serum levels reaching an alarming 192 μg/L among chrome plating workers [62]. Consequently, the highest documented PFAS levels occur in professional settings [6,23,63]. Firefighters face severe chronic exposure from AFFFs and protective gear, showing significantly elevated serum levels [6,32,63,64]. Workers in fluorochemical manufacturing [63,65] and chrome plating (where PFOS acts as a mist suppressant) [6,23,63] are exposed primarily via the inhalation of aerosols. Additionally, professional skiers and ski equipment technicians are at risk through the inhalation of fluorinated wax fumes [63], and textile industry workers face exposure during fabric impregnation processes [63,65].

## 4. Human Health Risk

Increasing human exposure underscores the urgent need to reassess not only legacy compounds, but also emerging alternatives that exhibit significant bioaccumulative properties [66]. Evidence mapping across more than 150 PFAS compounds highlights substantial gaps in current toxicological understanding and statistical modeling approaches, particularly regarding systemic human health effects [67]. Consequently, contemporary clinical and epidemiological assessments must move beyond reductionist, linear models and adopt a more holistic interpretation of toxicological and population-based data.

When interpreting these findings, a fundamental methodological distinction must be made between statistical association and biological causality. The majority of the human epidemiological data discussed herein derive from observational cross-sectional or case–control studies. While these robustly identify associations between elevated PFAS serum concentrations and adverse health outcomes, they are inherently limited in establishing definitive causal pathways due to potential unmeasured confounding factors, co-exposures, and the possibility of reverse causality.

### 4.1. Re-Evaluation of Carcinogenic Hypotheses

Early epidemiological studies contributed to a strong narrative regarding PFAS carcinogenicity; however, this paradigm is currently undergoing substantial revision. The landmark C8 Science Panel reported “probable links” between PFOA exposure and kidney cancer as well as thyroid disease, has been critically reassessed in light of improved exposure classification, confounder control, and methodological rigor. Recent analyses fail to confirm a consistent causal relationship in general populations [68]. Similarly, evidence linking PFASs to thyroid cancer remains inconclusive, with meta-analyses demonstrating high heterogeneity and inconsistent associations ranging from positive to null or even inverse relationships [69]. Notably, specific high-exposure cohorts, particularly in China, report associations between PFAS mixtures and increased risks of nodular goiter and papillary thyroid carcinoma [70].

### 4.2. Hepatocarcinogenesis and Systemic Cancers

A marked discrepancy exists between experimental and epidemiological findings regarding liver and gastrointestinal cancers. In vivo and in vitro studies consistently demonstrate that PFASs can induce hepatocellular proliferation and tumor formation at high doses [71]. However, human epidemiological evidence remains inconsistent. Studies on colorectal cancer report mixed outcomes, including harmful, null, and even potentially protective associations under specific conditions [72]. Prenatal exposure studies suggest limited and compound-specific effects on testicular cancer risk, with sulfonates associated with slightly increased risk and carboxylates with decreased risk [73]. Mechanistically, PFAS-related carcinogenesis appears to be largely indirect, mediated through disruptions in lipid metabolism, oxidative stress, and activation of pro-oncogenic pathways [74].

### 4.3. Liver and Kidney Injury Under Co-Exposure

In contrast to oncological inconsistencies, evidence for PFAS-induced organ toxicity is comparatively robust. Short-chain PFASs have been associated with elevated alanine aminotransferase levels, indicating early hepatic injury [75]. Multiple studies link PFAS exposure to the progression of non-alcoholic fatty liver disease (NAFLD), with sex-dependent differences showing greater susceptibility among women [76]. Both population-based and experimental studies confirm intrahepatic toxicity and steatosis associated with PFOA accumulation [77]. In pregnant women, PFAS exposure modulates liver function parameters, reflecting cumulative toxic effects [78]. Mixture exposure analyses reveal systemic biomarker perturbations, suggesting multi-organ suppression [79]. Advanced modeling approaches further indicate that combined exposure to PFASs and toxic metals contributes to chronic kidney disease through sustained oxidative stress mechanisms [80].

### 4.4. Sex Dimorphism in Metabolic Syndrome and Lipid Profiles

Cardiometabolic effects of PFASs are highly dependent on age, sex, and chemical structure. Contrary to earlier assumptions, many cross-sectional meta-analyses do not show a consistent association between major PFAS isomers and metabolic syndrome in the general population [81]. However, compound-specific associations have been observed: PFNA correlates with increased risk in adolescents, whereas PFHxS shows inverse associations in young adults [82]. Mixture exposure has been linked to metabolic syndrome predominantly in women [83]. In older populations, long-chain PFASs are associated with dysglycemia, reduced HDL levels, and increased waist circumference [84]. PFAS exposure also alters lipid metabolism in non-occupationally exposed populations [85], including associations with LDL cholesterol [86], thereby contributing to cardiovascular risk via lipid-mediated pathways [87]. Notably, dose–response relationships for type 2 diabetes often exhibit non-linear (parabolic) patterns [88], while uric acid mediates increased gout risk under inflammatory conditions [89].

### 4.5. Childhood Obesity and Metabolic Programming

Early-life exposure to PFASs influences adipocyte development and body composition trajectories. Alterations in adiposity have been observed in children, with clear sex-specific differences: prenatal exposure to PFAS mixtures is associated with decreased adiposity in boys but increased fat accumulation in girls [90,91]. In infancy, adiposity patterns reflected in weight-for-age z-scores (WAZ) are influenced by maternal PFAS burden [92]. Metabolomic studies further demonstrate disruptions in amino acid pathways linked to adipogenesis [93], confirming that metabolic dysregulation originates during fetal development [94].

### 4.6. Fetal Programming and Pregnancy Outcomes

The placenta represents a critical target of PFAS toxicity, mediating the relationship between prenatal exposure and later health outcomes [95]. Maternal exposure has been associated with accelerated epigenetic aging in cord blood [96]. Long-chain PFASs may reduce birth weight and alter placental hemodynamics [97], although these effects are modified by co-exposure to toxic metals [98]. Additional mechanisms include altered adipocytokine expression [99] and changes in anogenital distance [100]. Increased risks of congenital anomalies [101,102] and preterm birth [103] have also been reported. PFAS exposure disrupts maternal sleep architecture, contributing to systemic oxidative stress [104,105], and is implicated in gestational diabetes [106], particularly under modifying conditions such as infectious disease exposure [107]. Thyroid axis dysregulation within the maternal–fetal unit is well documented [108,109], while associations with hypertensive disorders of pregnancy remain heterogeneous across populations [110,111].

### 4.7. Gonadal Dysfunction and Infertility

PFAS exposure affects reproductive function in both sexes, though clinical manifestations vary. In women, PFASs are associated with altered ovarian function and increased risk of infertility and early menopause [112]. In vitro studies demonstrate receptor dysregulation in human granulosa cells [113], while uterine smooth muscle exhibits increased proliferative activity [114]. Clinically, higher PFOS levels correlate with increased prevalence of polycystic ovary syndrome [115]. In assisted reproduction, PFAS concentrations in follicular fluid are associated with reduced embryo quality and impaired cleavage rates [116,117]. In men, PFAS exposure is linked to decreased semen quality [118], suppression of Leydig cell markers and estradiol levels [119], and disruption of the blood–testis barrier [120]. Paternal exposure may also induce epigenetic alterations in sperm, affecting offspring transcriptomic profiles [121], with combined hepatotoxicity further exacerbating reproductive damage [122].

### 4.8. Neurodevelopment and Behavioral Outcomes

The neurotoxic effects of PFASs are characterized by substantial heterogeneity and analytical complexity. Some cohort studies report associations between PFOA exposure and increased risks of autism spectrum disorder (ASD), attention-deficit/hyperactivity disorder (ADHD), and developmental delays, often following non-linear dose–response relationships [123,124,125]. Conversely, inverse associations observed for PFAS mixtures are likely attributable to systematic bias rather than true neuroprotective effects [125]. Prenatal exposure has been linked to reduced cognitive performance in early childhood [126], although some studies report improved language outcomes and faster reaction times in adolescents [127]. Sleep disturbances may originate in utero, manifesting as increased nocturnal awakenings in infancy [128]. In adults, sex-specific patterns emerge, with inverse associations between PFAS exposure and anxiety in men, but strong positive associations with anxiety and depression in women [129].

### 4.9. Immune Function and Skeletal Effects

PFAS exposure induces immunosuppression, particularly at epithelial barriers such as the skin and mucosa [130]. Impaired adaptive immune responses and reduced vaccine efficacy, including diminished tetanus antibody production, represent critical public health concerns in exposed children [131]. These effects are accompanied by systemic inflammation and cytokine dysregulation mediated by macrophage activation [132]. While inhalation exposure increases asthma risk [133], paradoxical reductions in allergic symptoms have been observed in some infants, potentially due to prenatal immune modulation [134]. Additional systemic effects include telomere length alterations [135] and interference with vitamin D and folate metabolism [136]. Bone tissue responses are heterogeneous, with PFOS associated with decreased bone mineral density, whereas PFBS exposure has been linked to increased lumbar spine density [137,138]. Dental mineralization is similarly compound-specific [139]. Finally, PFAS exposure significantly alters the gut microbiome, reducing beneficial bacterial populations and increasing pathogenic taxa from early infancy [140].

### 4.10. Integrated Evidence Synthesis

Given the breadth and heterogeneity of PFAS-associated health outcomes across multiple organ systems, a structured synthesis of epidemiological and experimental evidence is presented in Table 5. This table integrates findings across metabolic, hepatic, endocrine, reproductive, neurodevelopmental, and immunological domains, highlighting both consistent patterns (e.g., hepatotoxicity, immunosuppression) and areas of substantial uncertainty (e.g., carcinogenicity, neurobehavioral outcomes).

### 4.11. Methodological Limitations in Exposure Assessment

A critical limitation in current PFAS epidemiology is the inherent risk of exposure misclassification. Many studies rely on single temporal spot samples (e.g., one-time blood draws) to estimate historical or chronic exposure, which may fail to accurately capture the cumulative toxicological burden. Furthermore, the interpretation of biomonitoring data is deeply confounded by the extensive variability in biomarkers. The biological half-lives of PFASs exhibit significant inter- and intra-individual differences influenced by genetics, age, and sex-specific excretion pathways—such as menstruation, lactation, and transplacental transfer—complicating accurate dose–response modeling.

## 5. Polar Regions as Indicator Environments for Contamination

Polar regions, due to their remote location and absence of local industrial emission sources and relatively pristine ecosystems, function as natural laboratories for observing the long-range transport of pollutants through the atmosphere and oceans [143,144]. The very presence of PFASs in Antarctic environments serves as direct evidence of their global distribution and provides an early warning of intensification of pollution, consistent with the One Health approach linking environmental, animal, and human health perspectives [3].

Indicator organisms such as penguins of the genus *Pygoscelis* allow for effective monitoring of polar ecosystem contamination through the analysis of PFAS presence in eggshells, feathers and guano [3]. Temporal trend analysis proves particularly valuable in this regard: a study of Adélie penguin (*Pygoscelis adeliae*) eggs spanning 1997–2021 demonstrated a decline in PFOS concentrations consistent with the timeline of international bans on its use, alongside a concurrent increase in long-chain PFCA concentrations, a group not yet subject to regulation during the study period [145]. These findings confirm the utility of polar environments as a tool for evaluating the effectiveness of global PFAS regulations, while simultaneously highlighting the need to update existing legal frameworks to address emerging contaminants and close the regulatory gaps that allow unregulated substitutes to perpetuate environmental contamination on a global scale.

## 6. Conclusions

The pervasive environmental contamination by per- and polyfluoroalkyl substances (PFASs) presents an unprecedented public health crisis driven by the extreme thermodynamic stability of the carbon–fluorine (C–F) bond, which renders these compounds highly recalcitrant to conventional remediation. Empirical evidence demonstrates that toxicological risk assessments must pivot from reductionist single-compound models toward holistic mixture and co-exposure paradigms. While observational associations regarding general population carcinogenicity exhibit statistical heterogeneity, the epidemiological and experimental evidence strongly suggests an association with systemic multi-organ toxicities—specifically intrahepatic steatosis, chronic kidney disease progression, and barrier-surface immunosuppression. However, given the reliance on observational study designs and the inherent variability in exposure biomarkers, these findings must be interpreted with methodological caution, strictly distinguishing between statistical correlation and biological causality. Furthermore, the industrial substitution of legacy long-chain congeners with short-chain alternatives has backfired; these homologues possess heightened hydrogeological mobility, facilitate long-range transport, and resist standard granular activated carbon adsorption. This management crisis is severely compounded by international regulatory fragmentation, where the sharp divergence between stringent US EPA maximum contaminant levels (4 ng/L for PFOA/PFOS) and broader European or Asian thresholds compromises unified risk mitigation across globalized supply chains.

To mitigate this transgenerational toxicological pressure, strategic interventions must prioritize class-based legislative frameworks aligning with the 2021 OECD expanded criteria, thereby eliminating the cycle of regrettable commercial substitutions. Concurrently, environmental engineering priorities must shift from purely segregative phase-transfer technologies toward advanced destructive modalities capable of complete C–F bond mineralization, such as electrochemical oxidation, non-thermal plasma, and supercritical water oxidation. Finally, future longitudinal cohorts must systematically integrate sex-specific metabolic dimorphisms and fetal epigenetic programming mechanisms, supported by targeted, high-resolution biomonitoring of critical occupational and pediatric populations.

## Figures and Tables

**Table 1:** Physicochemical properties of PFOA and PFOS based on PubChem data.

Property	PFOS	PFOA
Formula	C_8_HF_17_O_3_S	C_8_HF_15_O_2_
Molecular Weight	500.13 g/mol	414.07 g/mol
Water Solubility	~680 mg/L	9.5 g/L
Vapor Pressure	0.002 mmHg (20 °C)	0.525 mmHg (25 °C)
Log Kow	4.49	4.81

**Table 2:** Main PFAS applications and associated exposure routes.

Sector/Product	PFAS Type	Exposure Route	Ref.
AFFFs	PFOS, PFOA, precursors	Ingestion (water), dermal, inhalation	[6,13]
Cookware (Non-Stick)	PTFE, PFOA	Ingestion (migration)	[13,29]
Food Packaging	PFCAs, PFOS, fluorotelomers	Ingestion (migration)	[29,30]
Protective Masks	PFCAs, 6:2 FTOH	Inhalation, ingestion, dermal	[28]
Cosmetics	PTFE, PFCAs, fluorotelomers	Dermal, inhalation, ingestion	[9,13,31]
Impregnated Textiles	PFCAs, PFSAs, short-chain	Dermal, inhalation (dust)	[26,27,32]

**Table 3:** Comparison of regulatory limits for PFASs in drinking water.

Regulation	Substance/Group	Limit Value	Ref.
EFSA (2020)	Sum of 4 PFASs	4.4 ng/kg bw/week	[41]
US EPA (2024)	PFOA/PFOS	4 ng/L (MCL)	[39]
EU Directive 2020/2184	Total PFASs/sum of 20	500 ng/L/100 ng/L	[38]
Australia (2025)	PFOS/PFOA	8 ng/L/200 ng/L	[42]
Japan (MHLW)	Sum of PFOA + PFOS	50 ng/L	[43]

**Table 4:** Dermal absorption kinetics, permeability coefficients, and tissue retention of selected PFASs.

PFAS Compound	Carbon Chain Length	Dermal Absorbed Fraction (%)	Fraction Retained in Skin Tissue (%)	Permeability Coefficient (Papp or Kp)	Reference
PFPeA	C5	58.9 ± 1.9	17.3 ± 3.5	Papp = 4.33 × 10^−2^ cm/h	[52]
PFBS	C4	48.7 ± 4.2	19.6 ± 4.2	Papp = 1.41 × 10^−2^ cm/h	[52]
PFHxA	C6	36.4 ± 4.1	17.3 ± 3.4	Papp = 1.80 × 10^−2^ cm/h	[52]
PFOA	C8	13.5 ± 2.8	38.3 ± 5.4	Papp = 3.82 × 10^−3^ cm/h	[52]
PFOA	C8	1.6 *	Not reported	Kp = 4.4 × 10^−5^ cm/h	[54]
PFUnDA	C11	<LOD	66.5 ± 11.6	Not reported	[52]
PFNS	C9	<LOD	68.3 ± 19.6	Not reported	[52]

* Fractional absorption calculated under finite dose conditions recommended for human exposure assessments.

**Table 5:** Summary of epidemiological and experimental evidence on PFAS-associated health outcomes across major organ systems.

Health Domain	PFAS Compounds	Study Types/Cohorts	Key Findings	Ref.
Metabolic health and diabetes	PFOA, PFOS, PFNA, PFHxS, MeFOSAA, mixtures (C_mix)	Systematic reviews with meta-analyses; NHANES, STRIVE, Korean and Spanish cohorts	PFAS mixtures increase risk of type 2 diabetes (T2DM), with PFOS as a primary driver; PFOA shows a non-linear, parabolic dose–response relationship with T2DM; overall associations with metabolic syndrome (MetS) are inconsistent; age- and sex-specific associations are observed, especially for blood pressure, HbA1c, dyslipidemia, with stronger effects in women and adolescents (PFNA).	[81,82,83,88,89]
Lipid metabolism and cardiovascular system	PFOA, PFOS, Sm-PFOS, PFNA, PFDA, PFDeA, PFHxS, PFUA, PFUnDA, PFTrDA	Global meta-analyses; Jinan and PREDIMED-Plus cohorts; animal models	PFASs, especially PFOA, PFOS, PFNA, PFHxS, PFDA, PFDeA, and PFUA, show strong positive associations with total cholesterol (TC) and LDL in adults; effects are more evident in individuals over 40 years; TC and LDL mediate PFAS–CVD relationships; some mixture analyses show inverse or inconsistent associations with CVD.	[84,85,86,87]
Hepatotoxicity and liver function	PFOA, PFOS, PFNA, PFHxS, PFBS, PFDA, PFHpA, PFUnA, short- and long-chain mixtures	Pregnant cohorts (Guangxi Zhuang), STRIVE, rodent models, systematic reviews	There is consistent evidence of hepatotoxicity in humans and animals; exposure increases ALT, AST, GGT, and bilirubin; PFASs are associated with NAFLD progression and fibrosis, with stronger effects in women and non-obese individuals; high doses induce liver tumors in rodents, but human liver cancer evidence remains inconsistent.	[71,75,76,77,78,79,80,141]
Endocrine system and thyroid function	PFOA, PFOS, PFHxS, PFHpS, PFHpA, PFNA, PFDA, PFUnA, MeFOSAA, EtFOSAA	Project Viva, NHANES (youth), case–control studies (China)	Updated epidemiology does not support a causal link between PFOA and thyroid cancer or kidney disease; PFASs act as endocrine disruptors; prenatal exposure to mixtures lowers FT4I in mothers and T4 in male newborns; PFHxS and PFHpS are associated with nodular goiter, and PFHpA with papillary thyroid cancer.	[68,69,70,108,109]
Female reproductive system and fertility	PFOA, PFOS, PFNA, PFHxS, PFDeA, PFBS, PFDoA, PFTrDA, mixtures	PRISMA reviews; IVF/ART cohorts	Background exposure in the general population does not consistently reduce fertility, but high plasma and follicular-fluid PFAS levels in infertility patients reduce mature oocyte number, fertilization rates, and embryo quality; mechanisms include altered sphingolipid metabolism and steroidogenesis; higher levels are also associated with endometriosis, PCOS, delayed maturation, and earlier menopause.	[112,113,114,115,116,117]
Male reproductive system and semen quality	PFBS, PFuDA, PFTrDA, PFDoA, PFOS, PFOA, mixtures	Systematic epidemiological reviews; experimental mouse studies	PFAS mixtures may lower estradiol (E2) and the E2/TT ratio in young men, with PFuDA as a main driver; PFTrDA and PFDoA impair Leydig cell function by reducing INSL3; PFASs reduce semen quality, sperm DNA integrity, and may disrupt the blood–testis barrier and induce oxidative stress; paternal exposure can cause sperm methylation changes with long-term offspring effects.	[118,119,120,121,122]
Pregnancy and developmental toxicity	PFOA, PFOS, PFNA, PFDA, PFUA, PFBS, PFDoA, PFUnA, PDA, 9Cl-PF3ONS	Birth cohorts (Shanghai Birth Cohort, Sheyang, New Hampshire, LIFECODES); animal models	Prenatal exposure is associated with hypertensive disorders of pregnancy, preeclampsia, placental dysfunction, sleep disturbance in pregnancy (especially PFBS), congenital structural anomalies, miscarriage, and preterm birth; effects on birth weight are inconsistent and may be sex-specific; mixtures can increase anogenital distance and alter cord-blood adipokines.	[95,96,97,98,99,100,101,102,103,104,105,106,107,110,111]
Neurodevelopment and mental health	PFOA, PFHpA, PFUnDA, PFHxS, PFOS, PFDA, PFDoDA, PFNA	CHARGE, JECS, FLEHS, Maoming cohorts	Early exposure to PFOA and PFHpA has been linked to ASD, ADHD, and developmental delay in children, though findings are inconsistent; some cohorts report impaired early communication/problem-solving, while others report better selective attention or higher developmental scores; adult anxiety shows sex-specific patterns, with lower anxiety in men and higher anxiety in women; prenatal exposure is associated with infant sleep disturbances.	[123,124,125,126,127,128,129,142]
Immunotoxicity and immune system	PFOA, PFHxS, PFOS, PFNA, mixtures	Meta-analyses in children; California Teachers Study	PFASs reduce immune response to vaccination in children, including lower tetanus antibody titers; prenatal exposure to mixtures is associated with reduced lung function (FEV1 and FVC) in young adult males; in adult women, PFASs modify circulating inflammatory markers, including BAFF, TNFRII, IL2Ralpha, and sCD14.	[130,131,132,133,134]
Other systems (kidney, bone, teeth, vitamins)	PFOA, PFOS, PFBS, PFHpA, PFNA, PDA, mixtures	NHANES cross-sectional studies; prenatal cohorts; in vitro studies	PFAS mixtures are positively associated with gout risk in adults, mainly mediated by uric acid; PFOA is associated with chronic kidney disease and lower eGFR; PFOS increases oxidative stress markers in pregnant women; bone mineral density findings are inconsistent, with PFBS linked to enamel defects and PFHpA showing protective effects; PFASs also disrupt vitamin D and folate homeostasis.	[80,89,135,136,137,138,139,141]

## Data Availability

The original contributions presented in this study are included in the article. Further inquiries can be directed to the corresponding author.
